# Supplementary material for: Mechanistic multiscale modelling of energy metabolism in human astrocytes reveals the impact of morphology changes in Alzheimer’s Disease
Source: PLoS Comput Biol. 2023 Sep 20;19(9):e1011464. doi: 10.1371/journal.pcbi.1011464 (PMC10545114; doi:10.1371/journal.pcbi.1011464)
Supplement: S1 Text — S1: Stability analysis. S2: Spatial arrangements for 2D simulations in retangular shape. S3: Significance test for 2D realisation. S4: Local behaviour of ATP, ADP and PYR in maximum and minimum energised reaction site settings. S5: Simulating higher cellular activity in the perisynapses and more lactate efflux loci. S6: Spatial arrangement for 3D simulations. S7: Additional Figure for AD simulations. S8: Dimensionless system. S9: Detail on numerical methods for 2D simulations. S10: Details on numerical methods for 3D simulations. S11: Numerical Parameters. (PDF) [file pcbi.1011464.s001.pdf]

S1 Text: Mechanistic multiscale modelling of energy metabolism in human astrocytes reveals the impact of morphology changes in Alzheimer's Disease

Sofia Farina[1], Valérie Voorsluijs[2,3], Sonja Fixemer[2,4], David Bouvier[2,4,5], Susanne Claus[6], Mark Ellisman[7], Stéphane P.A. Bordas[1*], Alexander Skupin [2,3,7*]

S1: Sensitivity Analysis.

In order to quantify the uncertainty of the model parameters we execute a sensitivity analysis. We consider a rectangular domain rectangle of dimension $[0, l] \times [0, L]$, with width $l = 4\,\mu$m and a length $L = 140\,\mu$m, (as the one in the Section "Uniform and polarised distribution of reaction sites in a 177 rectangular domain"), with the influx of glucose in the right corner and outflux of lactate in the opposite corner. The parameters we test are the kinetic reaction rate coefficients, which for this analysis are homogeneously defined in the domain, as the classical law of mass action. To test the parameters, we run more than 1000 realisations where each rate constant is sorted from a normal distribution, details are shown in S1 Fig A.

We are interested in understanding each reaction rate coefficient's impact on the concentration average at the steady state. First, we visualise in S1 Fig B the relation between the reaction rate constants and the concentration of the different metabolites in average at the steady state plotting the realisations. This plot highlights that some concentrations are more susceptible to changes from a particular parameter. In particular, GLC presents a clear dependence on the rate constant of HXK. ATP and ADP, which are involved in more reactions, seem to be most affected by the rate constants of LDH, Mito and act. GLY is highly dependent on the rate coefficient of PYRK and PYR depends on the rate constant of LDH. Last, LAC presents a positive correlation with the rate constants of HXK and LDH and a negative correlation with act. Moreover, the variability of metabolite concentrations in the realisations is in

A

	Rate Constant	Variance
$K_{\text{HXK}}(\text{mM}^{-2}\text{s}^{-1})$	$6.19E-02$	0.025
$K_{\text{PYRK}}(\text{mM}^{-2}\text{s}^{-1})$	1.92	0.5
$K_{\text{LDH}}(\text{s}^{-1})$	$7.19E-01$	0.25
$K_{\text{Mito}}(\text{mM}^{-28}\text{s}^{-1})$	$8.13E-02$	0.025
$K_{\text{act}}(\text{s}^{-1})$	$1.69E-01$	0.025

B

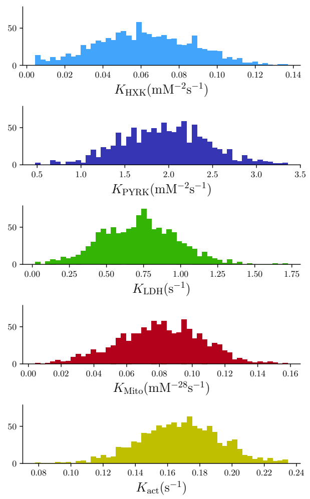

S1 Fig A. Reaction rate constant distributions for sensitivity analysis. A: The table described the rate coefficients for each reaction, the rate constant column shows the parameters used in the paper and the mean chosen for the normal distributions, and the variance column shows the respective variance. B: The histograms present the rate coefficient distributions for the realisations considered in the sensitivity analysis.

agreement with the experiments presented in the paper considering that in this experiment the reaction rate constants are homogeneous while in the paper we used spatial reaction rate. Furthermore, these results are related to complex metabolic analysis [1] where the flux control coefficients are non-other than the derivative of the concentration with respect to the rate constants, therefore the derivatives of S1 Fig B.

To quantify which rate constants have a significant influence on each concentration, we perform a variance-based sensitivity analysis computing the Sobol indices [2]. We calculate the first Sobol index and the total effect Sobol index. The first Sobol index,defined as $S_i = \frac{Var_{K_i}(\mathbb{E}[C_j|K_i])}{Var(C_j)}$, measures the direct effect each rate constant K_i has on the variance of the concentration C_j. The total effect Sobol index, $S_{T_i} = \frac{\mathbb{E}_{K_j,\, j\neq i}[Var(C_j|K_{j,\, j\neq i})]}{Var(C_j)}$, explain the importance of each reaction rate constants S_i in determining the output of the system, i.e. the metabolite concentrations C_j.

Sobol indices are meaningful if we have enough realisations, examining the average of metabolite concentration with respect to the number of realisations, in S1 Fig C, we see that with more than 1000 realisations, it does not show much oscillation in the

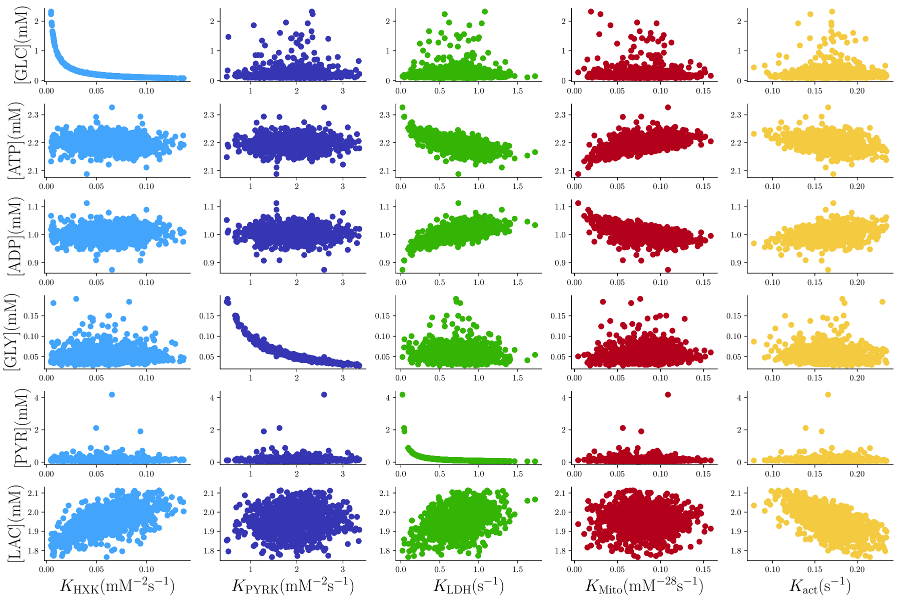

S1 Fig B. Impact of reaction rate coefficients variability on average metabolite concentrations at the steady state. These scatter plots present the relationship between the reaction rate coefficients and the concentration of the metabolites showing the behaviour of the realisations.

average implying that we have reached a sufficient number of samples. The histograms presented in S 1 Fig C show the Sobol indices confirming the observation we made based on S1 Fig B. In fact, the Sobol indices point out that GLC is only dependent on HXK rate constant and GLY on PYRK. These are trivial results since GLC and GLY amount can only be consumed by HXK and PYRK, respectively. On the other hand, the indices of ATP and ADP suggests more dependency. LDH affects the energetic level the most because it is the crucial reaction that competes for the substrate PYR. Not surprisingly the level of ATP and ADP are affected by the Mito and act, the first one producing most of the energy and the other one exchanging ATP in ADP. Interestingly, the Sobol indices of PYR shows dependency only on LDH, we suspect the reason why doesn't detect relation with Mito is the requirement of Mito reaction to have enough ADP to proceed while LDH is a linear reaction. Last LAC confirms the dependency noted in the previous Figure: HXK, LDH and act.

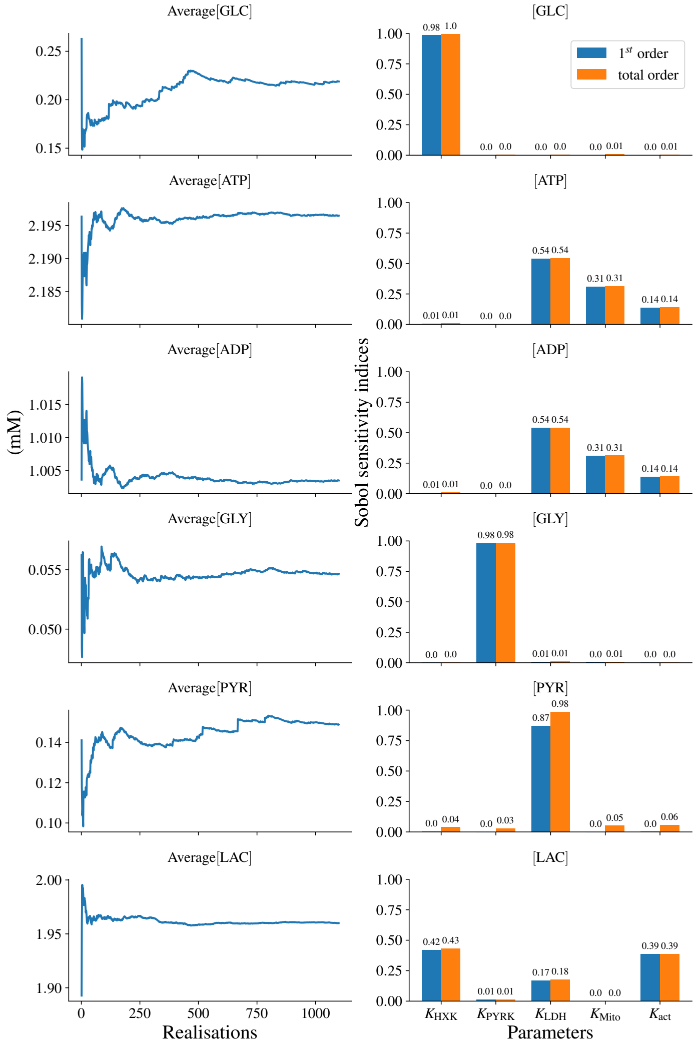

S1 Fig C. Quantify the reaction rate constants' impact on metabolites with Sobol indices. The right column shows the trend of the average metabolite concentrations varying the number of realisations, showing that the average gets stable enough to perform variance-based sensitivity analysis. The left column shows the first order and total effect Sobol indices for each metabolite.

S2: Spatial arrangements for 2D simulations in rectangular shape.

In the rectangular domain, we set up our simulation with ten reaction sites per reaction type. We define the entrance of GLC at the origin, the bottom left corner and the exits of LAC on the opposite vertex $(4, 140)\,\mu$m. The subregions of entrance and exits have been defined as the intersection of the rectangle and the circle with center the origin or the top right corner and radius $1.0\,\mu$m.

Reaction site distributions					
	Uniform		Polarised		Polarised $\log\mathcal{N}(2)$	
	x	y	x	y	x	y
HXK	$\mathcal{U}_{[0,l_1]}$	$\mathcal{U}_{[0,l_2]}$	$\mathcal{N}(\frac{l_1}{2},2)$	$\mathcal{N}(10,5)$	$\mathcal{N}(\frac{l_1}{2},2)$	$\mathcal{N}(10,5)$
PYRK	$\mathcal{U}_{[0,l_1]}$	$\mathcal{U}_{[0,l_2]}$	$\mathcal{N}(\frac{l_1}{2},2)$	$\mathcal{N}(10,5)$	$\mathcal{N}(\frac{l_1}{2},2)$	$\mathcal{N}(10,5)$
LDH	$\mathcal{U}_{[0,l_1]}$	$\mathcal{U}_{[0,l_2]}$	$\mathcal{N}(\frac{l_1}{2},2)$	$\mathcal{N}(l_2-10,5)$	$\mathcal{N}(\frac{l_1}{2},2)$	$\mathcal{N}(l_2-10,5)$
Mito	$\mathcal{U}_{[0,l_1]}$	$\mathcal{U}_{[0,l_2]}$	$\mathcal{N}(\frac{l_1}{2},2)$	$\mathcal{N}(10,5)$ or $\mathcal{U}[60,l_2]^*$	$\mathcal{N}(\frac{l_1}{2},2)$	$\log\mathcal{N}(2,2)$

S2 Table A. The table shows the distributions chosen for x and y coordinates for each metabolites for the simulations in the 2D rectangle for the Uniform, Polarised and Polarised $\log\mathcal{N}(2)$ cells. * indicates that we sorted the Mito sites from two distributions: six from the normal and four from the uniform. In this way, we ensure the probability of having four Mito sites on the top of the rectangle.

We present in S2 Table A, the different distributions used to define the x and y coordinates of the enzyme arrangements inside the 2D rectangle ($[0,l_1]\times[0,l_2]$). As presented in the main text, each setting has 10 reaction sites per reaction type. The uniform cells have all their sites sorted from a uniform distribution, noted $\mathcal{U}[a,b]$ covering the whole rectangular domain.

In polarised cells, we assumed the enzymes to be distributed according to normal distribution ($\mathcal{N}(m,\sigma')$, where m and σ' are the mean and standard deviation, respectively) or log-normal ($\log\mathcal{N}(m,\sigma')$) distributions. Close to the GLC influx we place HXK and PYRK reactions, using $\mathcal{N}(\frac{l_1}{2},2)$ and $\mathcal{N}(10,5)$ to select the x and y coordinates, respectively. The ten enzymes of LDH are located close to the LAC efflux with $(x,y)\in(\mathcal{N}(\frac{l_1}{2},2)),\mathcal{N}(l_2-10,5)$. For the ten mitochondria, we select the x coordinate as for the other reaction sites with a normal distribution $\mathcal{N}(\frac{l_1}{2},2)$. For polarized cells, one option consists in locating six reacting sites with y selected from a normal law $\mathcal{N}(10,5)$ and distributing the remaining four uniformly within the top part of the cell ($y>60$). We refer to this setting as the "Polarised" one. We also consider a different sampling where mitochondrial locations are distributed according to a log-normal law $\log\mathcal{N}(2.,2)$. We call this setting "Polarised $\log\mathcal{N}(2)$".

Typical polarised configurations are although lacking mitochondria in the middle part of the cell as shown in the corresponding figure of the main text.

S3: Significance test for 2D realisation

We run 200 realisations for each of these configurations (*i.e.* uniform, polarised and polarised $\log \mathcal{N}(2)$ for the purpose of statistical testing. In order to find out if the three types of spatial arrangements lead to statistically significant differences in the realisations, we perform a multiple comparison Holm-Bonferroni method since we consider simultaneously the distribution of the different concentrations. The Bonferroni method is applied to a parametric independent T-test to evaluate if there is a significant distance between the means of the concentrations of the three configurations and to a non-parametric Wilcoxon-Mann-Whitney test to verify if two statistical samples come from the same population. The results of the significance tests were presented in S3 Table A. When the $p-$value is smaller than 0.05 then the hypothesis of the tests is rejected, meaning that our samples describe different populations, which is mainly the case.

Holm-Bonferroni Method							
T test independent		[GLC]	[ATP]	[ADP]	[GLY]	[PYR]	[LAC]
Uniform	Polarised	0.0	0.0	0.0	0.093	0.0	0.0
Uniform	Polarised $\log \mathcal{N}(2)$	0.0	0.0	0.0	0.0	0.0	0.0
Polarised	Polarised $\log \mathcal{N}(2)$	0.0	0.0	0.0	0.0	0.598	0.0
Wilcoxon-Mann-Whitney test		[GLC]	[ATP]	[ADP]	[GLY]	[PYR]	[LAC]
Uniform	Polarised	0.0	0.0	0.0	0.0001	0.0	0.0
Uniform	Polarised $\log \mathcal{N}(2)$	0.0	0.0	0.0	0.0	0.0	0.0
Polarised	Polarised $\log \mathcal{N}(2)$	0.0	0.0	0.0	0.0	0.345	0.0

S3 Table A. $p-$values of the significance tests for Experiment 2. We used multiple comparisons Holm-Bonferroni Method on a parametric independent T-test and a non-parametric Wilcoxon-Mann-Whitney test with a significance threshold of $p-$value < 0.05.

S4: Local behaviour of ATP, ADP and PYR in maximum and minimum energised reaction site settings.

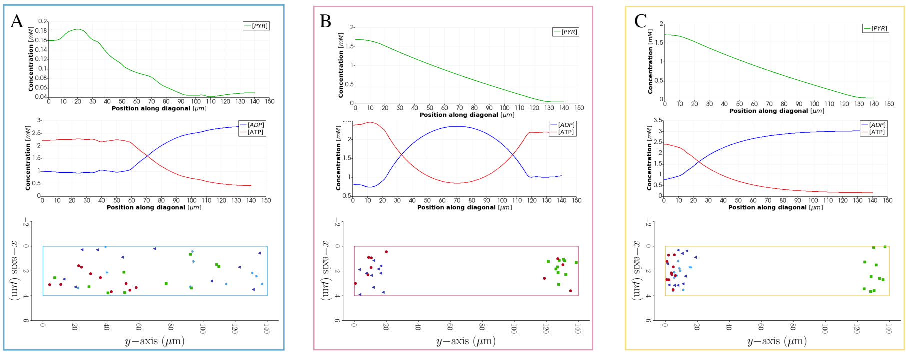

S4 Fig A. Spatial behaviour of ATP, ADP **and** PYR **in the less energised reaction site settings.** The behaviour of PYR, (top), ATP and ADP (middle) and the corresponding reaction rate configuration (bottom) for the less energised settings of the A: Uniform cells, B: Polarised cells C: Polarised lognormal cells.

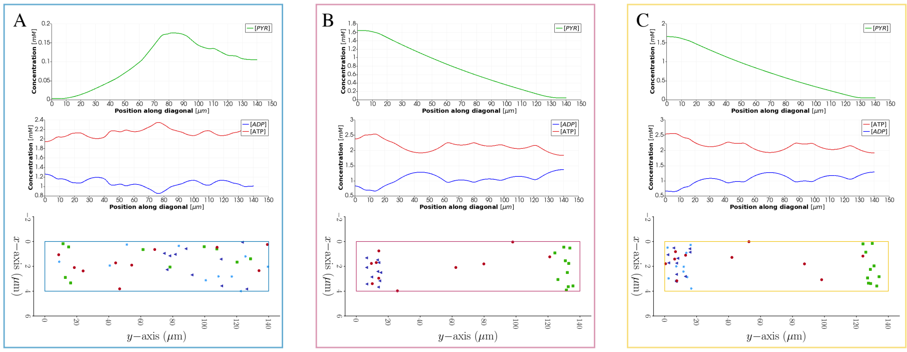

S4 Fig B. Spatial behaviour of ATP, ADP **and** PYR **in the more energised reaction site settings.** The behaviour of PYR, (top), ATP and ADP (middle) and the corresponding reaction rate configuration (bottom) for the more energised settings of the A: Uniform cells, B: Polarised cells C: Polarised lognormal cells.

S5: Simulating higher cellular activity in the perisynapses and more lactate efflux loci.

In the simulations presented in the manuscript the cellular activity was considered homogeneous inside the domain, and we kept this setting through the whole manuscript. However, it would be more right to define the cellular activity higher closer to the perisynaptic activity and lower in the endfeet of the astrocytes. In this section, we define the cellular activity inside the control and reactive astrocytic morphology using a Gaussian function tuned to simulate higher cellular activity further from the endfeet, as shown in S5 Fig AA- AB. Moreover, we consider more lactate export subregions, getting closer to reality, with 10 subregions for each cell (S5 Fig AA- AB).

In S5 Fig A C, we compare the average concentrations at each time step of these

simulations, labelled Control and Reactive lactate efflux 10 (C_{LE10} R_{LE10}, with the simulations presented in the main manuscript that here we re-labelled C_{LE4} R_{LE4} to focus on the different number of lactate export regions between the simulations. The results show that on average defining the cellular activity higher in the perisynapses does not impact the energy level of the cells, as confirmed by the histogram presenting the ATP : ADP (S5 Fig AD). On the other hand, adding lactate exports loci decrease the average lactate present inside the two cells with respect to the results in the manuscript and export more lactate (S5 Fig AE). The higher level of LAC exported in these simulations point to the fact that 1) more loci are able to export more because they act on the local concentration 2) the simpler shape of the control astrocyte supports better the surrounding neurons.

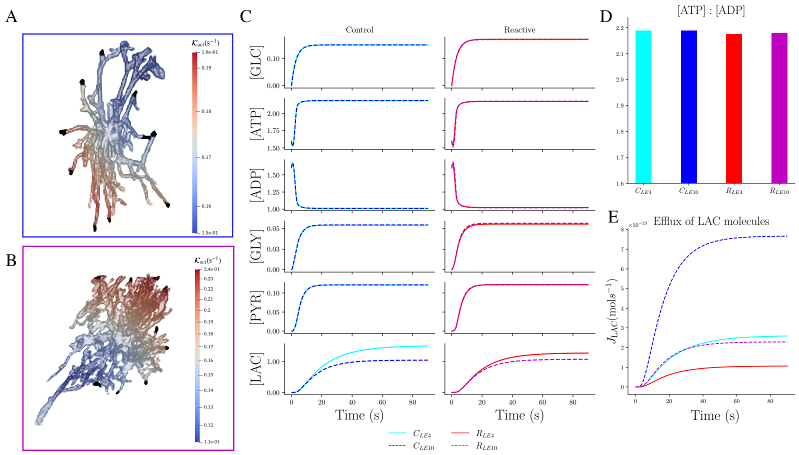

S5 Fig A. Metabolite dynamics in 3D astrocytes with physiological reaction site with ten lactate efflux loci and higher cellular activity in the perisynaptic regions. A: Control astrocyte (labelled: C_{LE10}) and B: reactive astrocyte (labelled: R_{LE10}) where the spatial reaction rate of cellular activity is simulated using a Gaussian function which is located close to the endfeet with variances tuned in order to obtain higher activity far from the endfeet. The 10 subregions where lactate is exported to neurons are highlighted in black. C: Average concentrations of metabolites evolving in time. The new simulations with healthy parameters consider the cellular activity located with a spatial reaction rate for control (blue) and reactive (magenta) and are compared with the results obtained with a homogeneous cellular activity (light blue and red). D-E: Comparison of the ATP:ADP ratio D: and valuation of the LAC exported E: for the homogeneous and spatially localised cellular activity in control and reactive astrocytes.

Investigating the results spatially, in S5 Fig B, we can appreciate the uneven effect

of cellular activity, higher close to the perisynapses (in the branches further from the endfeet) resulting in lower local ATP. Locally, the LAC export subregions are highlighted by lower local concentration of LAC.

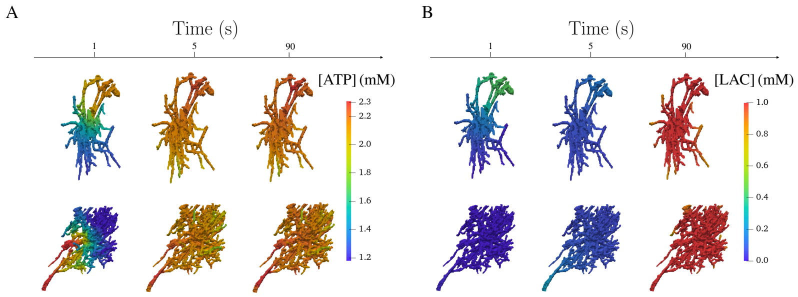

S5 Fig B. Spatial ATP **and** LAC **in 3D astrocytes at three time steps.** Spatial solution of A: ATP and B: LAC for C_{LE10} and R_{LE10} at 3 different time steps (t=1,5, 90). The cellular activity results higher in the branches located further from the end feet with a lower ATP concentration. The regions where lactate is exported show low local LAC levels.

S6: Spatial arrangement for 3D simulations.

We present in detail the settings of the simulation shown in Fig 6A- 6C of the main manuscript. For the control, C, and the reactive, R, LDH and PYRK sites were sorted by a uniform distribution defined in the boxes that contains the two astrocytes, see S6 Fig A. While HXK sites were sorted from a normal distribution centered in each Mito site and with variance $0.03 \times L$ where L is a dimensionless parameter (see below section S8). In particular, $1.38\,\mu$m for the control and $2.75\,\mu$m for R.

The polarised settings were arranged colocalising HXK and PYRK enzymes from a uniform distribution that cover the endfeet of the astrocyte ($\mathcal{U}_{[0,l_x]\times[y_1,l_y]\times[0,l_z]}$, where l_x, l_y, l_z are the dimensions of the box that contains the control astrocyte). LDH were sorted from the opposite side of the box containing the LAC export sub-regions of the astrocyte from another uniform distribution $\mathcal{U}_{[0,l_x]\times[0.,y_2]\times[0,l_z]}$. While the Mito were sorted from a log-normal distribution $\log\mathcal{N}(l_x, 0.64, l_z, 0.2)$ in the manner that colocalise HXK and PYRK.

Last, the three sub-regions chosen for the GLC entrance and the four LAC exits

were selected manually and they are defined as the intersection of the cellular morphology with a sphere with a radius of $1.0\,\mu$m.

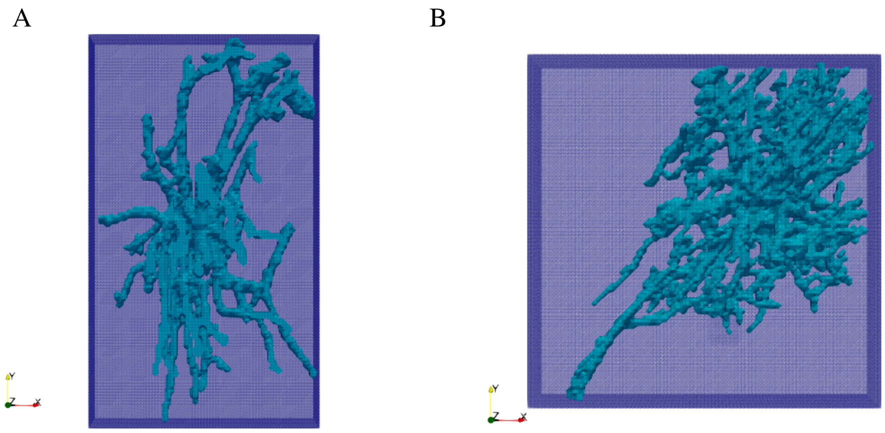

S6 Fig A. Astrocytic morphologies embedded into a finite element background mesh. A: control and B: reactive AD astrocytes are implicitly defined using a level set function and embedded in a structured background mesh. This is how we separated the finite element mesh from the geometries of the objects. Moreover, we used the dimensions of the background meshes to define the bounds of the uniform distributions to sort some of the reaction site centers.

S7: Additional Figure for AD simulations.

We present in S7 Fig A an extension of Fig 7 of the main manuscript, where we show the trajectories of the average concentrations of the metabolites. This plot highlights the behaviour of the system in response to different AD-related conditions. The systems reach the steady state in ≈ 50(s).

S8: Dimensionless system.

To obtain the dimensionless system from the RDS Eq. 6, we impose $[\bar{\mathrm{GLC}}] = \frac{[\mathrm{GLC}]}{\alpha}$, $[\bar{\mathrm{ATP}}] = \frac{[\mathrm{ATP}]}{A_{tot}}$, $[\bar{\mathrm{ADP}}] = \frac{[\mathrm{ADP}]}{A_{tot}}$, $[\bar{\mathrm{GLY}}] = \frac{[\mathrm{GLY}]}{\alpha}$, $[\bar{\mathrm{PYR}}] = \frac{[\mathrm{PYR}]}{\alpha}$, $[\bar{\mathrm{LAC}}] = \frac{[\mathrm{LAC}]}{\alpha}$, where A_{tot} is the total amount of ATP and ADP inside the cell and α a constant that we set to 0.16. The spatial dimensionless is $\bar{x} = \frac{x}{L}$ $\bar{y} = \frac{y}{L}$ and $\bar{z} = \frac{z}{L}$ where L is a

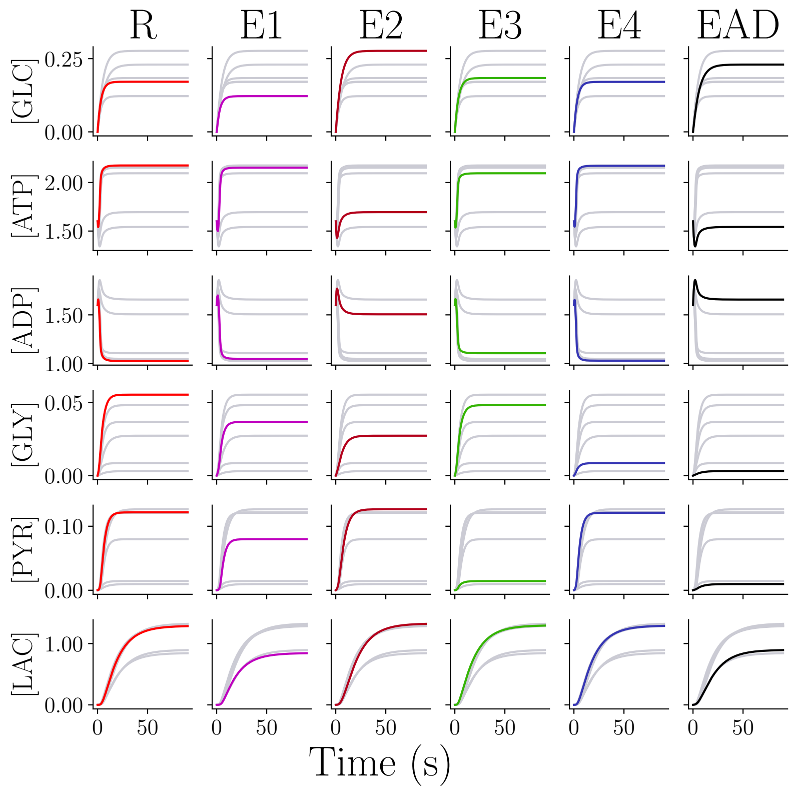

S7 Fig A. Effects of AD conditions on metabolite dynamics in 3D reactive astrocyte. Dynamics of the average concentration of each metabolites the simulations are solved inside the reactive astrocyte in AD with the setting presented in Fig 6 C of the main manuscript. R is the solution obtained with healthy parameters presented in Table 1 (red). E1 describes the deficiency of GLC uptake (magenta); E2, the mitochondria dysfunction (dark red); E3, the LDH overwork (green); E4, PYRK overwork (blue) and EAD, the four conditions combined (black).

parameter based on the volume of the astrocyte:

$$L = \frac{V}{V_\Omega}$$

where V is the real volume of the astrocyte segmented and V_Ω is the volume of the astrocyte in the dimensionless box with fixed x size of 1.0. We also define the dimensionless time parameter as $\bar{t} = \frac{t}{t_c}$ and we choose $t_c = \frac{L^2}{D_{\mathrm{GLC}}}$. So, we have that the dimensionless system is:

$$
\begin{cases}
\frac{\partial[\bar{\mathrm{GLC}}]}{\partial \bar{t}} = & \nabla^2[\bar{\mathrm{GLC}}] - \mathcal{K}_{\mathrm{HXK}}\beta_{[\bar{\mathrm{GLC}}]}[\bar{\mathrm{GLC}}][\bar{\mathrm{ATP}}]^2 + \delta_{[\bar{\mathrm{GLC}}]}J_{\mathrm{in}} \\
\frac{\partial[\bar{\mathrm{ATP}}]}{\partial \bar{t}} = & \frac{D_{[\mathrm{ATP}]}}{D_{[\mathrm{GLC}]}}\nabla^2[\bar{\mathrm{ATP}}] - 2\mathcal{K}_{\mathrm{HXK}}\beta_{[\bar{\mathrm{ATP}}]}[\bar{\mathrm{GLC}}][\bar{\mathrm{ATP}}]^2 + 2\mathcal{K}_{\mathrm{PYRK}}\gamma_{[\bar{\mathrm{ATP}}]}[\bar{\mathrm{ADP}}]^2[\bar{\mathrm{GLY}}] \\
& +28\mathcal{K}_{\mathrm{Mito}}\xi_{[\bar{\mathrm{ATP}}]}[\bar{\mathrm{PYR}}][\bar{\mathrm{ADP}}]^{28} - \mathcal{K}_{\mathrm{act}}\tau_{[\bar{\mathrm{ATP}}]}[\bar{\mathrm{ATP}}] \\
\frac{\partial[\bar{\mathrm{ADP}}]}{\partial \bar{t}} = & \frac{D_{[\mathrm{ADP}]}}{D_{[\mathrm{GLC}]}}\nabla^2[\bar{\mathrm{ADP}}] + 2\mathcal{K}_{\mathrm{HXK}}\beta_{[\bar{\mathrm{ADP}}]}[\bar{\mathrm{GLC}}][\bar{\mathrm{ATP}}]^2 - 2\mathcal{K}_{\mathrm{PYRK}}\gamma_{[\bar{\mathrm{ATP}}]}[\bar{\mathrm{ADP}}]^2[\bar{\mathrm{GLY}}] \\
& +\mathcal{K}_{Act}\tau_{[\bar{\mathrm{ADP}}]}[\bar{\mathrm{ATP}}] - 28\mathcal{K}_{\mathrm{Mito}}\xi_{[\bar{\mathrm{ADP}}]}[\bar{\mathrm{PYR}}][\bar{\mathrm{ADP}}]^{28} \\
\frac{\partial[\bar{\mathrm{GLY}}]}{\partial \bar{t}} = & \frac{D_{[\mathrm{GLY}]}}{D_{[\mathrm{GLC}]}}\nabla^2[\bar{\mathrm{GLY}}] + 2\mathcal{K}_{\mathrm{HXK}}\beta_{[\bar{\mathrm{GLY}}]}[\bar{\mathrm{GLC}}][\bar{\mathrm{ATP}}]^2 - \mathcal{K}_{\mathrm{PYRK}}\gamma_{[\bar{\mathrm{ATP}}]}[\bar{\mathrm{ADP}}]^2[\bar{\mathrm{GLY}}] \\
\frac{\partial[\bar{\mathrm{PYR}}]}{\partial \bar{t}} = & \frac{D_{[\mathrm{PYR}]}}{D_{[\mathrm{GLC}]}}\nabla^2[\bar{\mathrm{PYR}}] + \mathcal{K}_{\mathrm{PYRK}}\gamma_{[\bar{\mathrm{ATP}}]}[\bar{\mathrm{ADP}}]^2[\bar{\mathrm{GLY}}] - \mathcal{K}_{\mathrm{LDH}}\mu_{[\bar{\mathrm{PYR}}]}[\bar{\mathrm{PYR}}] \\
& -\mathcal{K}_{\mathrm{Mito}}\xi_{[\bar{\mathrm{PYR}}]}[\bar{\mathrm{PYR}}][\bar{\mathrm{ADP}}]^{28} \\
\frac{\partial[\bar{\mathrm{LAC}}]}{\partial \bar{t}} = & \frac{D_{[\mathrm{LAC}]}}{D_{[\mathrm{GLC}]}}\nabla^2[\bar{\mathrm{LAC}}] + \mathcal{K}_{\mathrm{LDH}}\mu_{[\bar{\mathrm{LAC}}]}[\bar{\mathrm{PYR}}] - \eta_{[\bar{\mathrm{LAC}}]}[\bar{\mathrm{LAC}}]
\end{cases}
\tag{1}
$$

where the dimensionless coefficients are shown in S8 Table A. In this way, we define a dimensionless system that depends only on the volume of the cell.

Dimensionless coefficients					
HXK	PYRK	LDH	Mito	act	
$\beta_{[\bar{\mathrm{GLC}}]} = t_c A_{\mathrm{tot}}^2$					$\delta_{[\bar{\mathrm{GLC}}]} = \frac{t_c}{\alpha}$
$\beta_{[\bar{\mathrm{ATP}}]} = t_c \alpha A_{\mathrm{tot}}$	$\gamma_{[\bar{\mathrm{ATP}}]} = A_{\mathrm{tot}} \alpha t_c$		$\xi_{[\bar{\mathrm{ATP}}]} = t_c \alpha A_{\mathrm{tot}}^{27}$	$\tau_{[\bar{\mathrm{ATP}}]} = t_c$	
$\beta_{[\bar{\mathrm{ADP}}]} = t_c \alpha A_{\mathrm{tot}}$	$\gamma_{[\bar{\mathrm{ADP}}]} = A_{\mathrm{tot}} \alpha t_c$		$\xi_{[\bar{\mathrm{ADP}}]} = t_c \alpha A_{\mathrm{tot}}^{27}$	$\tau_{[\bar{\mathrm{ATP}}]} = t_c$	
$\beta_{[\bar{\mathrm{GLY}}]} = t_c A_{\mathrm{tot}}^2$	$\gamma_{[\bar{\mathrm{GLY}}]} = A_{\mathrm{tot}}^2 t_c$				
	$\gamma_{[\bar{\mathrm{PYR}}]} = A_{\mathrm{tot}}^2 t_c$	$\mu_{[\bar{\mathrm{PYR}}]} = t_c$	$\xi_{[\bar{\mathrm{PYR}}]} = t_c A_{\mathrm{tot}}^{28}$		
		$\mu_{[\bar{\mathrm{LAC}}]} = t_c$			$\eta_{[\bar{\mathrm{LAC}}]} = \eta t_c$

S8 Table A. The table present the dimensionless coefficients for the dimensionless system 1.

S9: Detail on numerical methods for 2D simulations.

The 2D experiments were solved using standard finite element method (FEM) implemented in Python with the open source finite element solver DOLFIN from FEniCS [3, 4]. The domains are explicitly meshed using the package *mshr*, which generates a finite element mesh that conforms to the boundary of the domains. The solution of the weak problem is defined on the space of piece-wise Lagrange finite elements of degree one. We solve the non-linear equation using a Newton-Raphson scheme, where the Jacobian is calculated automatically by the automatic differentation

capabilities of UFL [5]. The linear system at each time step of the Newton-Raphson algorithm is solved using standard linear solvers from the *PETSc* library. For further details [6].

S10: Details on numerical methods for 3D simulations.

To solve the system on the 3D astrocytic domains, we use a cut finite element [7] approach able to deal with the complexity of the cellular geometry, as shown in [6]. The difference between FEM and CutFEM lies in the fact that FEM requires to generate a mesh conformed to the boundary of the domains. This can be a difficult task and CutFEM removes this need. In CutFEM, we describe the boundaries implicitly through a level set function [8, 9] that can be extracted from images. In particular, the level set function Φ is a scalar function that has negative values inside the domain, positive outside and zero on the boundary of the object. We obtained the level set, Φ, of the final segmented image f solving the following system in the domain B which is a three-dimensional box where we have mapped the image f:

$$\begin{cases} -\epsilon^2 \triangle \Phi + \Phi = f \quad \text{in}\, B \\ \nabla \Phi \cdot n = 0 \quad \text{on}\, \partial B \end{cases}$$

where ϵ is a smoothing parameter that we set to 0.001. This step smooths the boundary of the cells to reduce the mesh size and related computational complexity respectively. In S10 Fig A, we show the regular background mesh of finite elements covering the two astrocytic domains. To solve the weak formulation of the RDS using CutFEM, we define the fictitious domain as all the cells of the background mesh that have a non-zero intersection with the cellular domain (S10 Fig A). We apply a ghost-penalty stabilisation term [10] to all the edges that are intersected by the interface and all the edges that connect the intersected cells with the interior of the cellular domain. These stabilisation terms extend the solution from the physical domain Ω onto the fictitious domain. This is of fundamental importance to ensure the stability and accuracy of the numerical solution as the ghost-penalty stabilisation prevents ill-conditioning of the system matrices in case intersected cells contain very little of the cellular domain [7]. In

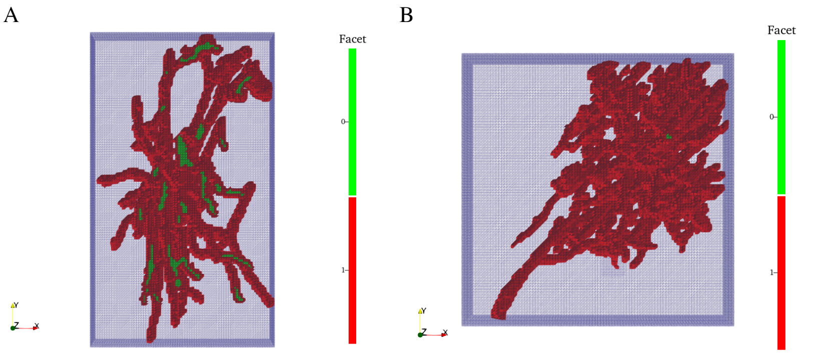

S10 Fig A. Fictitious domain and facet markers for the two astrocytic morphologies. Fictitious domain for the two astrocytic shapes A: control and B: reactive, defined as the non-zero intersection of the finite elements with the cellular domain. We apply the stabilization term to the facets marked in red.

2D Numerical parameters				
geometry	# cells	# dofs	max cell diameter	Δt
circle	88768	44856 *	0.93	0.25
star	51046	26164 *	0.77	0.25
rectangle	25298	13207*	0.33	0.17

S11 Table A. The table presents the numerical parameters used in the 2D experiments for the three domains: circle, star and rectangle. We show the number of cells in the finite element mesh, the number of degrees of freedom (dofs), the maximum diameter of the finite element cell and the time step used for the time discretisation. * In the table the number of dofs refers to one subspace. The total number of dofs for all the six subspaces is 269136 for the circle, 156984 for the star and 79242 for the rectangle.

this case, the linear system arising in the numerical experiments is solved using a direct ($MUMPS$) solver.

For further reading we address the reader to our previous work [6] and alternative proposed for enriched FEM [11–14].

S11: Numerical Parameters.

The parameters used for the 2D experiments are presented in S11 Table A and for the 3D experiments in S11 Table B. The penalty parameter γ used to ensure stability on the cut cells is set to 0.1. Convergence studies were done extensively in the 2-dimensional experiments.

3D Numerical parameters					
astrocyte	# cells in bg mesh	# cells on Ω	# dofs	max cell diameter	Δt
control	1000008	158938	37742	0.022	0.08
reactive	1301760	162579	39143	0.018	0.026

S11 Table B. The table presents the numerical parameters used in the 3D experiments for the control and reactive astrocytes. We show the number of cells in the background finite element mesh, the number of cells covering the astrocyte domains Ω, the number of degrees of freedom (dofs), the maximum diameter of the finite element cell and the time step used for the time discretisation. All parameters are dimensionless 1 and chosen based on the original astrocytic volume.

References

1. Heinrich R, Schuster S. The regulation of cellular systems. Springer Science & Business Media; 2012.

2. Hauseux P, Hale JS, Cotin S, Bordas SP. Quantifying the uncertainty in a hyperelastic soft tissue model with stochastic parameters. Applied Mathematical Modelling. 2018;62:86–102. doi:10.1016/j.apm.2018.04.021.

3. Logg A, Mardal KA, Wells G. Automated solution of differential equations by the finite element method: The FEniCS book. Heidelberg: Springer Science & Business Media; 2012.

4. Alnæs MS, Blechta J, Hake J, Johansson A, Kehlet B, Logg A, et al. The FEniCS project version 1.5. Archive of Numerical Software. 2015;3(100):9–23. doi:10.11588/ans.2015.100.20553.

5. Alnæs MS, Logg A, Ølgaard KB, Rognes ME, Wells GN. Unified form language: A domain-specific language for weak formulations of partial differential equations. ACM Transactions on Mathematical Software (TOMS). 2014;40(2):1–37. doi:10.1145/2566630.

6. Farina S, Claus S, Hale JS, Skupin A, Bordas SP. A cut finite element method for spatially resolved energy metabolism models in complex neuro-cell morphologies with minimal remeshing. Advanced Modeling and Simulation in Engineering Sciences. 2021;8(1):1–32.

7. Burman E, Claus S, Hansbo P, Larson MG, Massing A. CutFEM: discretizing geometry and partial differential equations. International Journal for Numerical Methods in Engineering. 2015;104(7):472–501. doi:10.1002/nme.4823.

8. Osher S, Sethian JA. Fronts propagating with curvature-dependent speed: algorithms based on Hamilton-Jacobi formulations. Journal of computational physics. 1988;79(1):12–49. doi:10.1016/0021-9991(88)90002-2.

9. Sethian JA. Level set methods and fast marching methods: evolving interfaces in computational geometry, fluid mechanics, computer vision, and materials science. Cambridge: Cambridge university press; 1999.

10. Burman E. Ghost penalty. Comptes Rendus Mathematique. 2010;348(21-22):1217–1220. doi:10.1016/j.crma.2010.10.006.

11. Babuška I, Melenk JM. The partition of unity method. International journal for numerical methods in engineering. 1997;40(4):727–758. doi:10.1002/(SICI)1097-0207(19970228)40:4¡727::AID-NME86¿3.0.CO;2-N.

12. Bordas S, Menk A. Xfem: The Extended Finite Element Method. John Wiley; 2014.

13. Agathos K, Chatzi E, Bordas SP, Talaslidis D. A well-conditioned and optimally convergent XFEM for 3D linear elastic fracture. International Journal for Numerical Methods in Engineering. 2016;105(9):643–677. doi:10.1002/nme.4982.

14. Agathos K, Bordas SP, Chatzi E. Improving the conditioning of XFEM/GFEM for fracture mechanics problems through enrichment quasi-orthogonalization. Computer Methods in Applied Mechanics and Engineering. 2019;346:1051–1073. doi:10.1016/j.cma.2018.08.007.
