# Supplementary material for: Mechanistic multiscale modelling of energy metabolism in human astrocytes reveals the impact of morphology changes in Alzheimer’s Disease
Source: PLoS Comput Biol. 2023 Sep 20;19(9):e1011464. doi: 10.1371/journal.pcbi.1011464 (PMC10545114; doi:10.1371/journal.pcbi.1011464)
Supplement: Attachment — Submitted filename: Response_To_Reviewers_Farina_Skupin.pdf [file pcbi.1011464.s014.pdf]

Dear Hugues, dear reviewers,

Thank you very much for your solid evaluation of our manuscript and your constructive criticism.

Based on your feedback, we have adapted and extended the manuscript to address your questions and comments. In general, we followed the suggestion of the reviewers where we still kept the overall simplifying assumptions since it allows for model validation and establishment but have included some more specific scenarios, in particular in respect to spatially inhomogeneous cellular activity.

Following the suggestion of Reviewer 3 we have changed the title to: "Mechanistic multiscale modelling of energy metabolism in human astrocytes reveals the impact of morphology changes in Alzheimer's Disease"

Please find below our point-to-point responses to the raised questions and explanations to the ms adaptations.

## Detailed Responses to Questions

**Comments to the Authors:**
**Please note here if the review is uploaded as an attachment.**

**Reviewer #1**: The manuscript by Sofia Farina and colleagues describes their modeling research focusing on astroglial energy metabolism in the context of neurodegenerative diseases, when astrocytes are hypothesized to loose their ability to sustain neurons with lactate.
The authors describe a minimalistic lumped model of cell metabolism and look at steady-state average metabolite concentrations depending on spatial arrangement of the four modeled enzymatic complexes or mechanisms, namely hexokinase (HXK), pyruvatkinase (PRK), lactate dehydrogenase (LDH), and oxydative phosphorilation in mitochondria (Mito). They start with arguably over-simplistic spatial profiles and proceed to realistic astrocyte morphologies extracted from experimental data.

While the approach is indeed original and provides interesting insights, one has to comment on several issues with the manuscript in its current form.

** General remarks and model organization

– The assumption that cellular activity should occur homogeneously inside the domains

(p. 9, line 165) is not sufficiently grounded; one can expect e.g. that perisynaptic processes can spend more energy restoring ionic gradients to fuel neurotransmitter uptake.

We agree with Reviewer 1 that the consumption of ATP is non-homogeneous inside the cell and is specifically higher closer to the perisynaptic processes [Pellerin 1994, Winkler 2017]. The main focus of our presented work was however on the spatiotemporal organization of the main metabolic energy-generating pathways thus privileging glycolysis, mitochondrial activity and lactate dehydrogenase. In fact, the aim of the first experiment in the circular domain was to study the interplay of spatial location on these main reactions without localizing also the cellular activity. This simplification of homogeneously distributing the cellular activity allowed us to focus on the effect of mitochondria's location without another free parameter.

Moreover, during the preparation of the manuscript, we also ran some simulations where the cellular activity was localized using the spatial reaction rates in the rectangular simulations. We sorted the location of the cellular activity sites using a uniform distribution that covered the whole rectangular domain.
In these tests locating the cellular activity generated some extreme configurations (for example, when cellular activity sites were clustered) which were leading to a very low average ATP: ADP due to the high localized concentration gradient of these two substrates.

Another reason for the choice of a homogeneous cellular activity is that the system with inhomogeneous cellular activity, in dependence on the spatial configuration, sometimes did not reach a steady state since GLY can accumulate, as exemplified for a specific configuration (Figure Rebuttal 1A) by the metabolite concentration dynamics shown in Figure Rebuttal 1B. Plotting the spatial variations in ATP, ADP and GLY concentrations along the diagonal of the rectangle (Figure Rebuttal 1C-E) highlights not only that ATP and ADP dynamics generate regions with high gradients (as mentioned above) but also that GLY is locally "exploding" where glycolysis is located. As shown in Figure Rebuttal 1D ADP is locally depleted and PYRK can thus not proceed. While this is per se also an interesting observation, the focus of the systematic analysis was on the impact of spatial coupling on the core metabolic pathways, and we therefore used a spatially homogenous cellular activity for the screening approach.

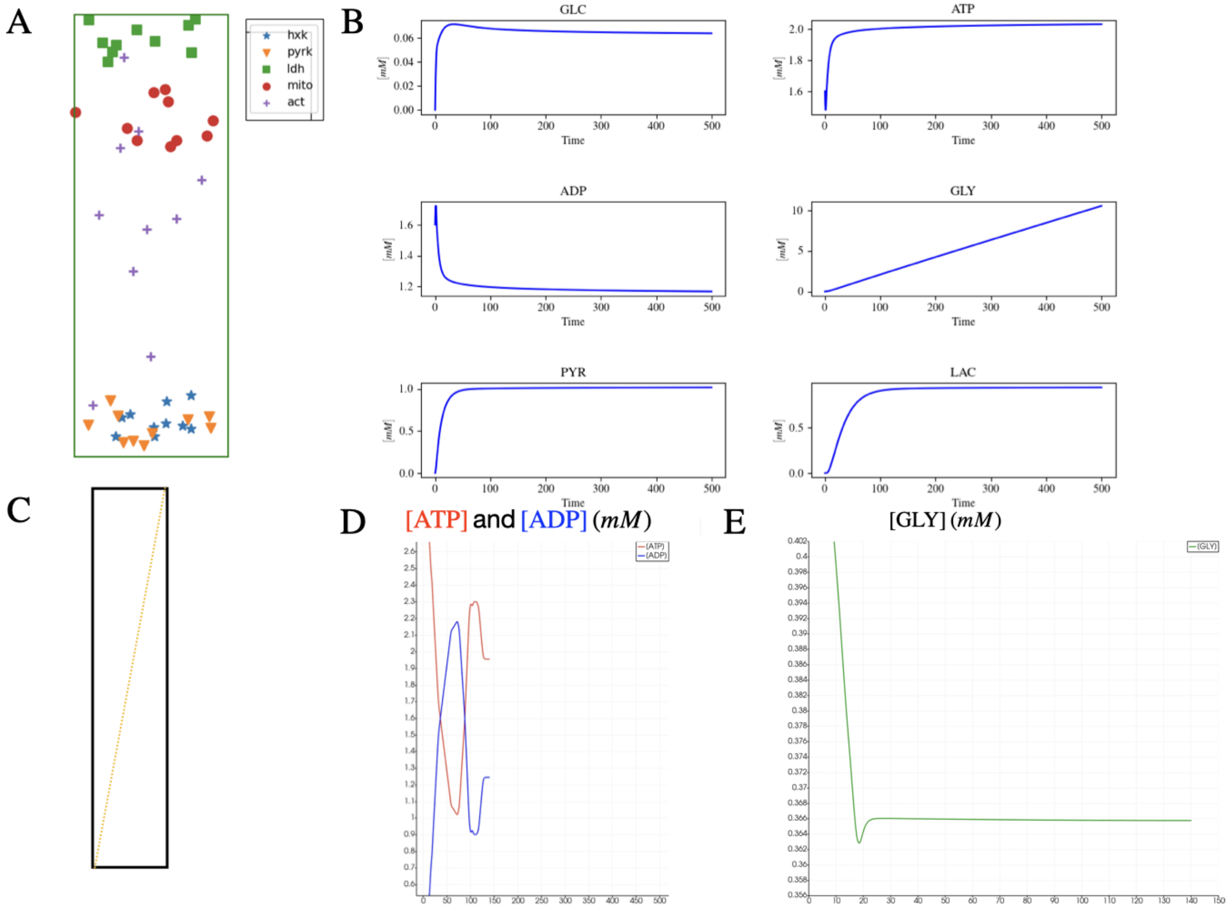


**Rebuttal Figure 1** A) Spatial arrangement of chemical reactions where cellular activity was localized in 10 reaction sites (magenta crosses) defined by a uniform distribution covering the whole rectangular domain. B) Average concentration of each metabolite as a function of time. C) The diagonal visualizes the 1d domain considered for the spatial behaviour of ATP, ADP and GLY concentrations in panels D and E. D,E) Concentration of ATP, ADP (D) and GLY (E) reached at the last simulation time step along the diagonal of the rectangle highlighted in (C).

Finally, we addressed the comment of Referee 1 regarding a refined definition of cellular activity in the perisynaptic regions. We ran two simulations in the control and reactive astrocytes considering a higher cellular activity at the perisynapses (i.e., at the tip of the branches) and included these simulations in the Supplementary Information as Figure 6. For this approach, we used a Gaussian function to simulate a lower cellular activity density function close to the end feet and higher activity on the branches (Figure Rebuttal 2 (A-B)) where the overall (integrated) activity remained the same as in the homogenous case. In these simulations, we also considered more subregions at which lactate (LAC) is exported addressing another remark of Reviewer 1 (see below). The results in Figure Rebuttal 2 (C-D) highlight that this definition of a spatially inhomogeneous cellular activity is leading to a slight change in the spatial concentration profiles but does not impact the

average concentration of metabolites and therefore does not change the overall conclusions of our work.

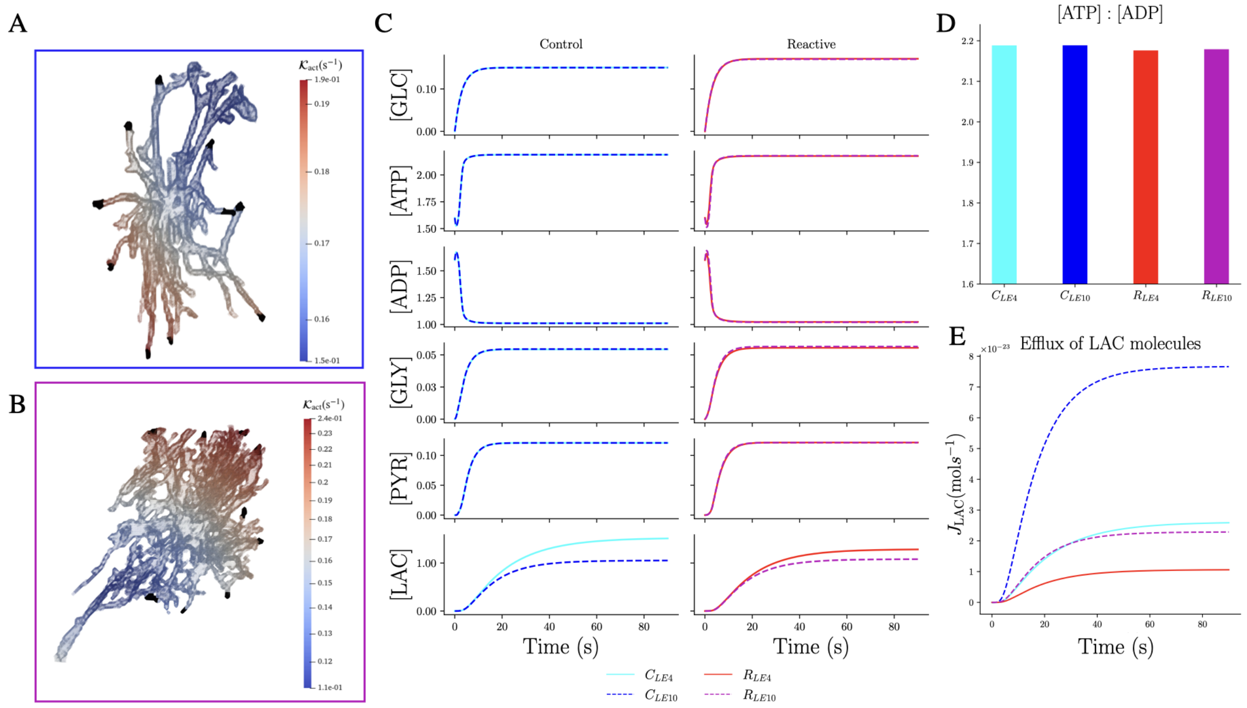


**Figure Rebuttal 2** A) New parametrisation of Control astrocyte (C_LE10) and B) reactive astrocyte (R_LE10) with 10 instead of 4 lactate export sites where the spatial reaction rate of cellular activity is simulated using a Gaussian function which is located close to the endfeet with variances tuned in order to obtain higher activity at the perisynapse far from the endfeet. The 10 subregions where lactate is exported to neurons are highlighted in black. C) Average concentrations of metabolites evolving in time. The new simulations with healthy parameters consider the cellular activity located with a spatial reaction rate for control (blue) and reactive (magenta) are compared with the results obtained with a homogeneous cellular activity (light blue and red from Fig. 6D of the ms) do not exhibit big differences except for lactate (LAC). D-E) Comparison of the ATP:ADP ratio (D) and valuation of the exported LAC (E) for the homogeneous and spatially localized cellular activity in control and reactive astrocytes.

For the model establishment, we thus focus in the present manuscript on a homogenously distributed cellular activity. However, we added a sentence (l. 110-112) to indicate to the readers that homogeneous cellular activity is an oversimplified assumption and to point the readers to the supplementary material to these simulations (l. 305-308).

For more extreme inhomogeneous arrangements, also the average concentrations may differ since the system may not reach the steady-state as in the scenario discussed above in relation to Rebutal Figure 1. However, this is beyond the scope of the present ms and will be investigated in more detail in follow-up studies.

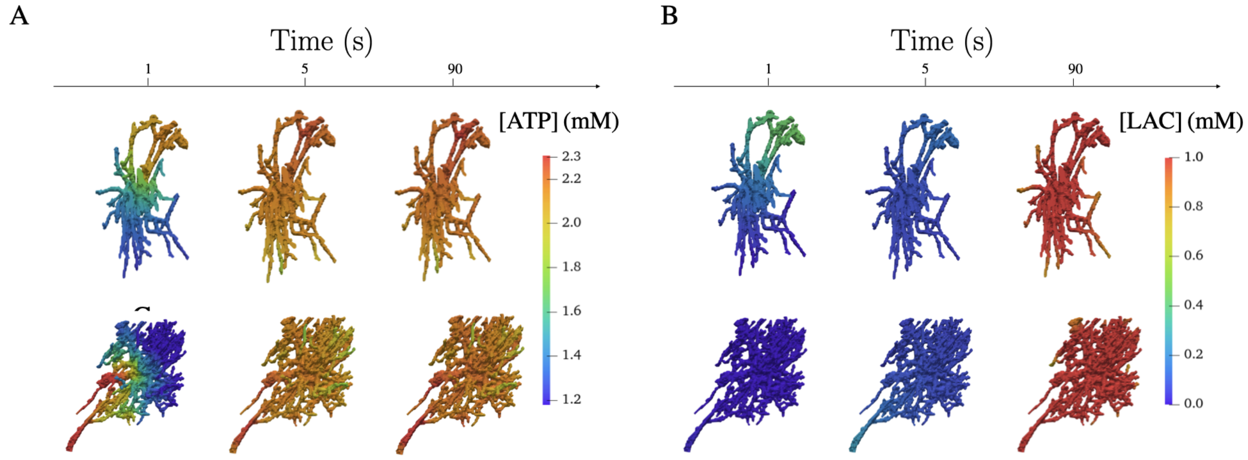


**Figure Rebuttal 3 A)** ATP and **B)** LAC spatially resolved for C_LE10 (top line) and R_LE10 (bottom line) at 3 different time steps (t={1,5, 90}). The 3D spatial concentration shows the effect of cellular activity refined in the perisynaptic regions with a lower concentration of ATP in the branches further from the end feet. The higher number of LAC export regions is also marked by lower local LAC concentration in the spatial results.

– Narrow spatial confinements of GLC influx and LAC outflux have to be justified. While GLC influx can be speculated to be confined to astro. endfoot, LAC outflux seems more likely to be sprinkled over multiple perisynaptic loci.

In the first step towards a realistic geometrical simulation of metabolism, we decided to consider a simplified polarization of input/output (entrance/exit) of metabolites from the opposite sides of the cell. We then decided to be consistent throughout our manuscript and ran simulations with these settings from the 2D to the 3D.

Based on Reviewer 1 comment that lactate exits should be spread in more loci where perisynaptic activity is happening, we ran the simulations with more lactate subregions (10 each) in both the control and reactive astrocyte as explained above and shown in Figure Rebuttal 2 (C-E). While the integrated export rate remained constant, adding more loci of lactate export leads to higher overall export of lactate. This is further indicating the impact of spatial arrangement since it demonstrates that distributing lactate export to more regions increases the overall export since local concentration gradients are smaller.

The simple morphology of the control astrocyte exports more lactate, suggesting that the control astrocyte is supporting the neuron properly, while the reactive one is less efficient.

In conclusion, we do agree with the comment of Referee 1, and in this regard, we added a sentence in the manuscript (l. 313-319 and l. 488-490) pointing to our complementary

simulations presented here in Figure Rebuttal 2 and 3 and which are now included in Supplementary Information Figure 6.

– what dictates the choice of log-normal distribution of enzymatic complexes or mitochondria?

Normal and log-normal distributions were used in the screen complementarily to simulate and analyse the effect of the assumed polarized spatial arrangement of enzymes in astrocytes. The skewed and asymmetrical shape of log-normal distributions allowed us to generate unbiased and automatically spatial arrangements in which enzymes'/mitochondria's locations are slightly more spread along the cell in comparison with the normal distributions (as shown for example by the position of mitochondria generated by normal *vs* log-normal distributions in Figure 4B). A log-normal distribution of mitochondria was used to simulate the colocalization of glycolysis with mitochondrial activity while allowing for residual mitochondria in the middle of the cell and acting as "relays" to LDH on the other cell side. This configuration was generated to investigate the cell outcome when pyruvate is "forced" to encounter first mitochondria and LDH only after a longer diffusion process, which is the point of the next comment of Reviewer 1.

We agree with Referee 1 that the choice of the statistical distributions could be better explained, and we clarified it now in the text in lines 210-215.

– while at first a competition for pyruvate between Mito and LDH is mentioned (p. 11, line 199), it is somehow transformed into Mito-PYRK competition in p. 14, line 251, going as far as a suggestion that PYRK "inhibits mitochondrial activity". This is counter-intuitive, because PYRK is the source of substrate for Mito.

We thank the reviewer to point to this inconsistency. Indeed, our model is focusing on the competition between Mito and LDH for the substrate PYR produced by PYRK reaction. Therefore, in line 251 we made the confusing statement that PYRK "inhibits mitochondrial activity". In fact, PYR is well produced by clustering glycolysis in the bottom of the rectangle (see Figure Rebuttal 2-3 B-E and also Figure 3 in the ms). However, the Mito reaction requires 1 molecule of PYR and 28 ADP to produce 28 ATP, and the local depletion of ADP leads to an accumulation of PYR. The lack of ADP substrate and the resulting slow mitochondrial activity are shown in **Figures Rebuttal 4 and 5** for the less and most energised cells, respectively.

Looking at the local concentrations of ATP and ADP along the diagonal of the rectangle highlights that the key issue in the minimum ATP settings lies in the fact that Mito clustering creates regions with high ATP or ADP gradients.

We have now clarified the corresponding description by removing the confusing line 251 from the previous version of the manuscript and added a more detailed discussion about the fundamental role of the highly variable local concentrations of ADP (l. 267-271). In this respect, the corresponding two figures were also included in Supplementary Information Figure 4.

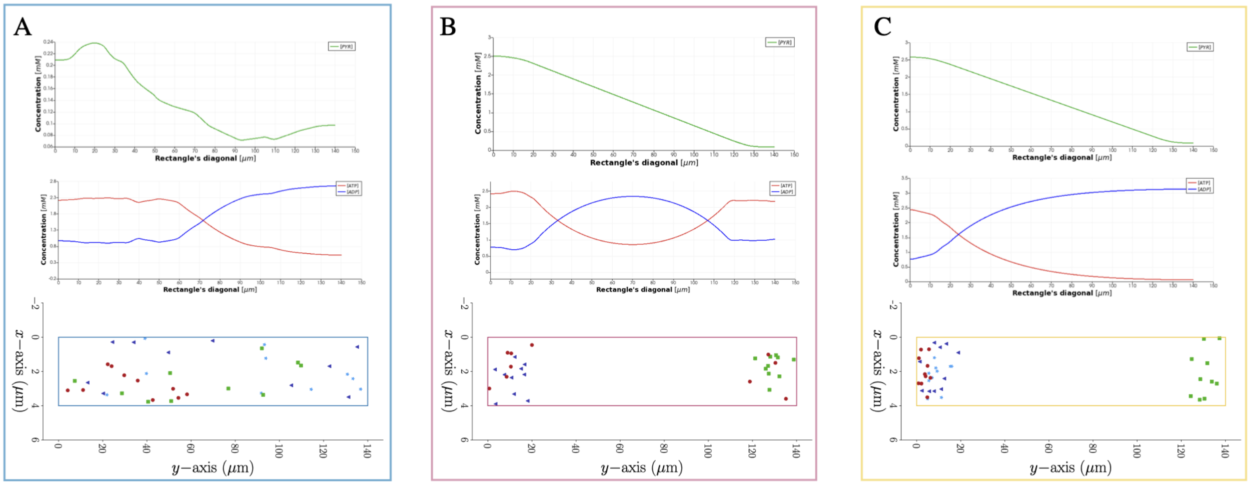


**Figure Rebuttal 4**: The panels show the local behavior along the diagonal of the rectangle of PYR (top), ATP and ADP (middle) and the respective reaction site configurations Uniform A), Polarised B) and LogNormal C) (bottom). These configurations are the ones leading to the less energized cells.

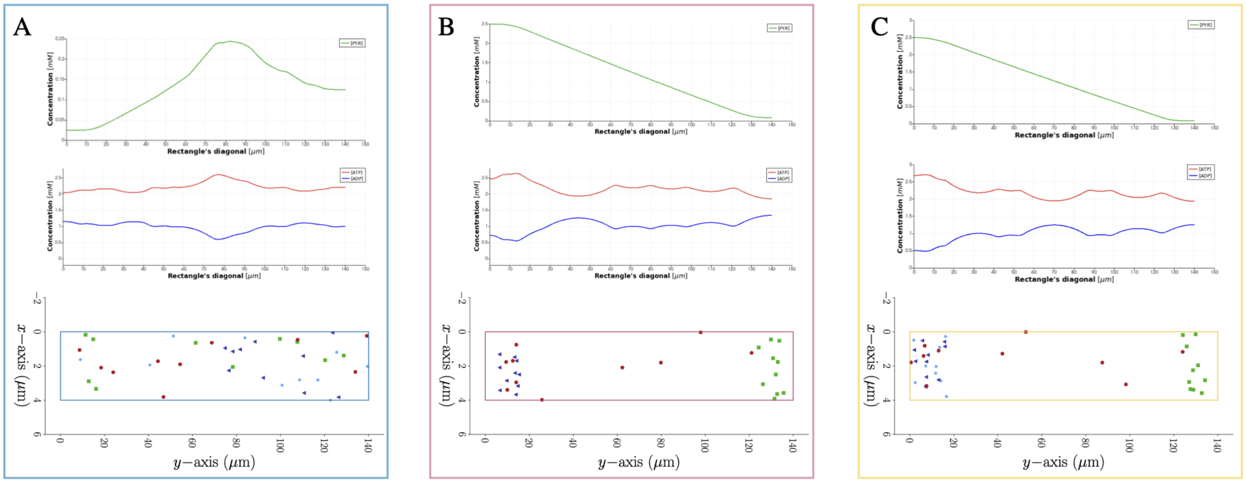


**Figure Rebuttal 5**: The panels show the local behavior along the diagonal of the rectangle of PYR (top), ATP and ADP (middle) and the respective reaction site configurations Uniform A),

Polarised B) and LogNormal C) (bottom). These configurations are the ones leading to the most energised cells.

– It is further unclear why the lowest levels of ATP are observed in the arrangements with all mitochondria grouped near HXK and PYRK. Shouldn't a placement of Mito closer to the site of substrate production enhance ATP production? An alternative explanation of the observed results could be in the line that if the mitochondria are grouped, then any runaway PYR molecule will have a chance to be converted to LAC at the far end of the elongated cell, while spreading out mitochondria along the cell axis will reduce the change for PYR to escape its oxyphospho fate.

As mentioned in response to the previous point, the production of ATP is linked to the availability of the substrate ADP, which is locally lacking in this configuration, as shown above in **Figures Rebuttal 4-5** and now detailed in the manuscript (l. 267-271) and shown in Supporting information Figure 4.

– The fact that high ATP levels inhibits glycolysis, discussed in the ATP:ADP-related papers (e.g. Maldonado, Lemaster, Mitochondrion 2014) in the context of Warburg pathway is not accounted for in the model, but it can be rather important for the outcome.

We agree with the comment of Reviewer 1 but the aim of the present ms was to investigate the impact of the spatial arrangement and to establish a first multiscale model able to investigate integrated energy pathways in a spatial manner. Hence, refining the mathematical model by incorporating enzyme regulation is an important next step to further investigate the metabolic behaviour of astrocytes but is beyond the proof-of-concept scope of the ms on spatiotemporally metabolic dynamics described by inhomogeneous reaction-diffusion systems in complex geometries. However, we added a sentence in the discussion section to point to the readers that this is something that clearly needs to be considered in further work (l. 486-88).

– The justification for the spatial arrangements in Figs. 3-4, especially the artificial second cluster of mitochondria near the LDH-enriched site in the "polarized" arrangement should be spelled out more explicitly.

We agree with Referee 1 that some of the spatial arrangements might appear arbitrary, but the aim of the simulations from Figs. 3-4 was not to reproduce realistic configurations. Instead, the role of those simulations is to test mechanistic hypotheses using limit cases – in this case, focusing on PYR consumption and diffusion, on the one hand, and the

spatial distribution of mitochondria, on the other hand. Determining the exact contribution of each hypothesis when several mechanisms are at play is very challenging with physiological arrangements. Using extreme (and hence artificially appearing) configurations is a way to validate a mechanism while avoiding the perturbations attributed to competing processes.

We clarified that notion in the manuscript with two additional sentences (l. 193-197).

– The observation (and explanation of) that non-reactive astrocyte exports more LAC than the more ramified reactive one is counterintuitive. This looks like noting that a less ramified pipeline system will deliver more water to a town than a more ramified one due to longer pathway of water to each household. It is easy to imagine that the more ramified astrocyte will have more LAC outlets to tap lactate to the tissue and will export more of it, so it's a question of the number of export regions.

Our simulations consider the same number of lactate regions for the two morphologies, leading to the conclusion that a simpler pipeline would deliver more lactate than a more complex pipeline. This is also confirmed in the new simulations we ran in **Figure Rebuttal 2-3**. However, we agree with Referee 1 that the reality is far more complex. The number of loci may vary from astrocyte to astrocyte and between the 'healthy' and the reactive state, and also the flux intensity may also increase, as well as the production of LAC by different levels of LDH. In future investigations, it will be interesting to assess the LDH abundance and to evaluate where the lactate transporters are - following the next comment of Reviewer 1.

– Concerning the spatial arrangement of LAC export sites. First, just four sites of LAC export per astrocyte seems like a severe underestimate. Second, I would suggest to address IHC images of LAC transporters in astroglial membrane (e.g. how uniform vs clustered they are).

We have addressed the number of lactate export sites in the previous answers above by additional simulations. As stated above, we agree that future investigations considering IHC images of LAC transporters in the astroglial membrane will be an important next step. For the here used samples, the staining is unfortunately not applicable anymore. In any case, we have added a corresponding sentence at l. 490-493.

– Overall, I would suggest to shorten the artificial template part of the research (e.g. by dropping the circle and star arrangements entirely) to emphasize the really relevant part with experiment-based morphologies. For example, by comparing more than one cells

from each group. This would also allow for a more clear description of and justification for the metabolic challenges simulated in the AD case. Additionally, the boundary between the simulation and physiological predictions for real cells seems just a bit too thin in the text allowing for statements like “The only cell that reaches a critical unhealthy state is the EAD condition”, “When cells lack GLC, they become more egoistic and produce more ATP”, “single AD characteristic does not lead to an unhealthy cell” etc. I would advocate for a more clear distinction between the (arguably, simplistic) simulation and the physiological implications from the simulation results.

While we appreciate the reviewer’s comment, one focus of the paper is to establish the spatiotemporal model by a proof-of-concept application. Thus, the manuscript aims to lay the foundation of the 3D simulation of metabolism in physiologically realistic in silico astrocytes. Therefore, we believe that displaying our 2D experiments of increasing complexity and number of reaction sites shows to the reader that geometries and reaction site locations are indeed crucial to the metabolic system output - a finding that is not yet well established in the scientific community where it is widely believed that diffusion on small spatial scales smear out and have no effect. Our findings clearly show systematically that this is not the case - specifically in complex geometries. Furthermore, 3D simulations are more computationally expensive than 2D simulations and the supplementary dimension increases the number of possible configurations, which makes systematic analyses, such as in Fig. 2, unnecessarily demanding.

Our established framework is easily transferable to other 3D astrocyte images and therefore sets a basis for future investigations in which we will address the raised questions in more 3D morphologies.

We believe that we have made a clear distinction in the ms between simplistic simulation and physiological, by highlighting for example, the parameters choices (L. 58-59); the choice of 2D simplified geometries (L.62-63); the choice of the size of the 2D domains (L.100); the justification for the choice of “Location 1” (L 118-120) and for the rectangular shape as an astrocytic branch (L. 185). Following one of the previous comments of Reviewer 1, we are now highlighting that polarized settings are not physiologically meaningful (L.192-196); that the 3D simulations’ physiological choices have been justified (L. 185), like for the mitochondria (L.289), and the AD conditions (L.325-333). Moreover, we incorporated the suggestions of Reviewer 1 to make the reader more aware of refining the cellular activity definition and exporting lactate in more subregions (L.303-309).

** Figures and results representation

– Fig. 1:
- [p. B,C]: what is the physical meaning and what are the units for the colorbar?

Panels B and C represent the areas with metabolic activity, with dark blue and yellow corresponding to less and more activity, respectively. Mathematically, the plotted function is $\sum_{i=1} \mathcal{G}(\mathbf{x}_i,\sigma_i) \quad i=\{\HXK,\PYRK,\Mito,\LDH\}$, where a single position x_i (and corresponding variance) is allocated per reaction. Given the definition of \mathcal{G}(\mathbf{x}_i,\sigma_i), the associated units are $\mu m^{-2}$.

We addressed the question of Reviewer 1 by adding labels and units to the colorbar in panels B and C of Figure 1.

- [p. B,C]: why are some of the x-axis and y-axis values negative (the template also being not centered)?

We decided to locate the GLC influx at the origin (0,0) of the axes. Since the circle has a radius 70 \mu m, the centre of the circle is, by construction, located at (36.05, 60) \mu m and some parts of the domain have negative x and y coordinates.

- [Caption]: may be it’s better to denote different spatial organizations as Arrangement 1 and Control instead of Location 1 and control?

We appreciate the Reviewer's suggestion and changed the name to Arrangement 1 and Control.

- [general]: what justifies combination of a pair of mechanisms in a single vertex? i.e. why not make 4 vertices for each of the complexes?

Co-locating two reactions on one vertex allowed us to study the local competition between reactions, which is not of the same intensity if each reaction is located on its own vertex.

– Fig. 3: to increase clarity, it’s would probably help to plot the distributions with empty background, in a “stair” style or alpha channel. Otherwise some parts of the distributions are occluded.

We used a bar-stacked plot which is a bar-type histogram where multiple data are stacked on top of each other. We tried other graphical representations, which were less efficient and not kept for the submission.

To clarify the interpretation of Fig. 3A, we added in the caption that the data are stacked on top of each other to help the reader better understand the plot.

– Fig. 4.
- It is unclear, what exactly is plotted in panel C and how it's related to spatial arrangements in panel D.

We clarify the relationship in the text l.272-276 and in the caption of the figure.

- Caption: "mitochondria activation" should be "mitochondria activity"

This is indeed a typo, which has been corrected.

– Fig. 5. The reconstruction of astrocyte morphologies seems imperfect, for example a large chunk of the endfoot from protoplasmic astrocyte appears to be missing (and it's unclear where's the endfoot in the AD astrocyte at all).

We thank Referee 1 for his/her feedback and have improved Figure 5. We have added arrows to help the reader visualise the end foot and changed the caption.

– Fig. 7. [panel A]: connecting lines have no meaning, please consider to remove them

We agree with Referee 1 that meaningless lines can be confusing for the reader. The lines in Figure 7A have been removed.

** References to literature
- There is no year or year is poorly formated: Refs. 7, 11, 22, 31

Thank you for prompting to this. The corresponding references have been changed.

- Ref 19 (Almeida et al PNAS 2001) is cited in support of the notion that ATP:ADP ratio in healthy cells should be > 1. However, this point is not made in the publication.

The reference has been changed to Tantama et al. 2013 (https://www.nature.com/articles/ncomms3550) who developed a biosensor to measure the intracellular level of ATP:ADP ratio in mammalian living cells. In particular, Fig. 3 of this reference shows how ATP:ADP ratios respond to glucose perturbation in astrocytes and these results are in agreement with the decrease in ATP:ADP observed in E1.

**Reviewer #2**: This manuscript presents a mechanistic model of energy metabolism in spatially resolved implementation considering idealized as well as realistic morphology of the cell (astrocyte). It is well written and should be of interest to the audience of the journal. As the modelling is complex and the numerical implementation involving a number of assumptions, I do believe that the clarity could be improved by providing more details on the following:

1) There are various sources of uncertainty - such as code uncertainty, parameter uncertainty, model discrepancy, and observation error - inherent to any computational model analysis. Prior to undertaking model-based inference it is important to consider such uncertainties and quantify as many as possible. I would recommend that the authors at least comment on this and ideally provide some uncertainty estimates for example, parameter sensitivity measures.

We agree with Reviewer 2 that modelling comes with a number of sources of uncertainty and uncertainty quantification is fundamental in computational models - in particular in computational precision medicine.

Thus, an important part of our research is to focus on developing uncertainty quantification tools for biomedicine and biomechanics [Urcun 2022, Eftimie 2023]. When creating a model, we have three main sources of errors: the model error, the discretization error and the numerical error.

The model error is involved in answering the question: “Are we solving the right problem?” The answer to this question is dependent on the quantity of interest which is to be understood and predicted by the model. The model error can be generated by multiple factors: incorrect biological assumptions, parameter choice, geometry, boundary, and initial conditions etc. Regarding these factors throughout the manuscript, we added explanations to justify our proposed model and assumptions. For example, discussions about the biological assumption (also following the suggestions of the reviewer at l. 76-77, 110-112), refining the model (l. 480-493) or about refining the astrocytic morphology (l 493-503) were added in the revised ms.

Concerning the discretization error linked to solving the mathematical model numerically, we added more information on the convergence by providing now details of refining the mesh and the time step of our simulation in response to Reviewer 3 (see below for details). Furthermore, in the discussion, we now point to developing a real-time estimation error to ensure the accuracy of discretization error (l. 496-499).

In conclusion, a modeler must ensure that both the modeling and discretization error are under control. This is usually done iteratively as any model change will lead to necessary modifications in the discretization choice as well.

As mentioned in the discussion, our model can be further improved. Yet, we validated the current model with steady-state values in the physiological range of astrocytes based on the literature, which indicates its ability to predict steady-state values properly. Finally, following Referee 2 suggestions, we include now a sensitivity analysis on the reaction rate constant parameters which is included in **Supplementary Information 1** and added a discussion on this point in the Discussion (l.496-499).

2) The choice for modelling metabolism by considering a rather coarse representation of metabolic reactions could be further justified.

We agree with Reviewer 2 that the kinetics of the modelled metabolic reactions is quite simplified. Notably, glycolysis is reduced to two key steps (HXK and PYRK) based on the rate-limiting and irreversible steps of glycolysis. As explained above in relation to Reviewer 1 comments, we decided to base our reaction-diffusion equations on a simplified version of metabolic kinetics in favour of keeping a high resolution of the complex astrocytic shape. Incorporating more details about the kinetics of metabolism, enzyme regulation or even cell compartmentalisation is a challenge that we started to address in a follow-up project.

We have added a few lines when the kinetic model is introduced to elaborate on the aforementioned modelling choices and priorities (l.76-77, l. 84 and l. 88).

3) The choices of uniform, normal and log-normal distributions for the reaction sites also need to be justified in more detail. Why these particular distributions? How realistic is this?

We thank Referee 2 for this remark, which was also made by Referee 1. We addressed this remark in the manuscript by justifying our reasoning in more detail (l.193-195). See also response to Reviewer 1 above.

4) I am not convinced in the following statement (page 13, lines 219-222): "Interestingly, the Polarised log N (2) configuration exhibits a very wide range for both concentrations with significantly different average values also in comparison with the Polarised configuration indicating the importance of mitochondrial distribution." How does this

indicate "the importance of mitochondria distribution" and why? Could you please elaborate.

We agree with Referee 2 that the sentence on page 13 lines 219-222 was rather vague and we rephrased it to make it clearer (l.236-240).

5) The choice of no-flux boundary conditions used when simulating the reaction-diffusion system needs to be justified. How biologically realistic is this assumption? Why is it acceptable?

We agree with Referee 2 that a justification for the choice of boundary conditions was lacking. We addressed this gap by adding a few words in the manuscript (l. 543-544). In brief, for the modelled species the cellular membrane is not permeable, and they have therefore to be transported actively through the membrane by transporter molecules. Moreover, this choice is consistent with previous metabolic models, such as [Aubert & Costalat 2005, Aubert & Magistretti 2005, Cloutier 2009, Jolivet 2009, Berry 2018, and others] who are also considering no flux boundary conditions and defining transport processes (and hence reaction rate) for the exchanges with the extracellular space (import of glucose, export of lactate). We added a line in the model description to justify this choice (l.544).

6) Typo on page 30, line 554 - "we discretise..." --> "We discretise..."

We thank Referee 2 for spotting this typo, which has been fixed.

**Reviewer #3**: First and foremost, let me congratulate the authors on a very well performed and presented study. The introduction is nicely written, clear and convincing. I especially appreciate the authors statement: "The underlying assumption that diffusion and reaction rates of metabolism are large enough to smear out spatial aspects are challenged by the complex morphology of astrocytes and an increasing amount of evidence for relocation of enzymes and other reaction sites in different conditions". The numerical experiments are well performed and results clearly described.

Major comments

I strongly suggest that

* The authors revisit the title. The current title, especially the "indicates morphological effects" is unnecessary unclear/weak.

We thank the Reviewer for this recommendation and changed the title to:
"Mechanistic multiscale modelling of energy metabolism in human astrocytes reveals the impact of morphology changes in Alzheimer's Disease"

* The authors revisit the last sentence of the Author summary. As in the case of the title, I find the phrase "fundamental to ensure robustness by buffering effect" unclear.

We rephrase that sentence in the author summary paragraph to: “The findings emphasize the importance of the spatial arrangement of metabolic reaction sites for the metabolic dynamics, which is further emphasised in the intricate structures of astrocytes where their morphological changes can be crucial for facilitating metabolic dysfunctions.”

* The authors considers whether Table 2 should be included as is, and whether the numbers are accurate. As is, the signal-to-noise ratio seems low. The table could be moved to the Supplementary, or the non-zero values described in the text.

Thank you for this recommendation. The values in Table 2 were obtained using the Python package statsmodels and reported exactly the values given by the Python function with the same digits.
The Table has been moved into Supplementary Information 2, and in the main manuscript, we added a description of the results (l.220-228).

* The authors include an estimate/numerical experiment relating to the accuracy of the numerical approximations for image-based geometries in Results. In the Supplementary, the authors state that "Convergence study were done extensively in the 2 dimensional experiments." I would encourage the authors to provide more detail. Furthermore, the question of how numerically accurate/converged/resolved the reported results from the image-based geometries are remains. I would encourage the authors to address this vital question.

We thank the reviewer for the vital suggestion which we took up and provide now more details about our analysis in the revised version of the ms.

First, considering the PDE model defining the chemical reaction by the classic law of mass action (therefore not using our spatial reaction rates), we verified that the average concentrations converge to the associated ODE system. Moreover, in our previous work [Farina et al. 2020], we verified the convergence of the system using an asymptotic solution of the ODEs and limiting the amount of glucose that entered the cell and no

lactate efflux. In this way, the concentration mass was conserved and allowed for direct validation..

Furthermore, the convergence study was run on the circle with all the reaction sites located in the center (as in the configuration in Figure 1 B of the manuscript). Since it is not possible to solve the PDE analytically, we use the average concentration of the metabolites at the steady state as the quantity of interest to verify the convergence. We run 10 simulations refining the mesh (from N=30 to N=170 which is the input parameter of the FEniCS mesh generator). First, we verified that the more we refined the mesh the more the average concentration was converging. Indeed, from **Figure Rebuttal 6** (below), we can appreciate that except for the first two meshes, which are too coarse, the other meshes reached convergence in the average steady state concentrations allowing us to claim that we have reached the convergence of the system. For this purpose, we used the finest mesh as reference and compute in **Table Rebuttal 1,** the relative error. In the simulations presented in the manuscript we used N=150 and from **Table Rebuttal 1** it is evident that the relative error is below 10^{-4} with the exception of LAC with the highest error of the order of 10^{-3} due to the defined efflux regions.

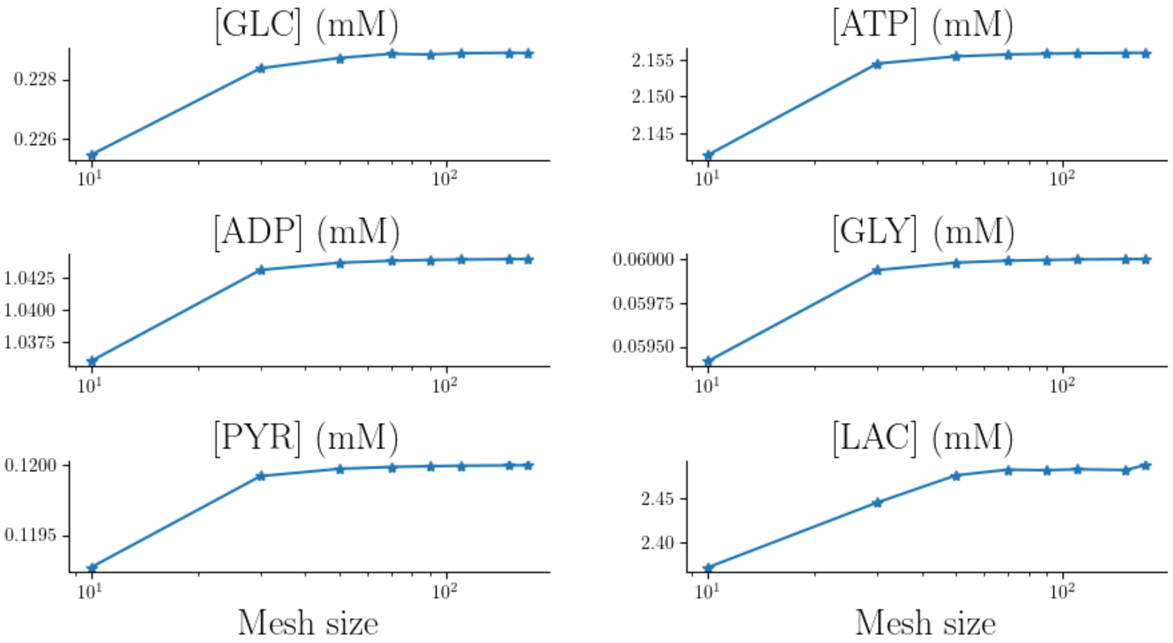


**Figure Rebuttal 6** The x-axis shows the considered mesh sizes in log-scale, precisely N={10,20,50,70,90,110,150,170} and y-axis the metabolite average concentrations at steady state.

| Mesh Size | [GLC]($T$) | [ATP]($T$) | [ADP]($T$) | [GLY]($T$) | [PYR]($T$) | [LAC]($T$) |
|---|---|---|---|---|---|---|
| 10 | 1.49e-02 | 6.41e-03 | 7.63e-03 | 9.67e-03 | 6.01e-03 | 4.70e-02 |
| 30 | 2.25e-03 | 6.74e-04 | 8.20e-04 | 1.05e-03 | 6.39e-04 | 1.71e-02 |
| 50 | 7.29e-04 | 2.27e-04 | 2.78e-04 | 3.41e-04 | 2.10e-04 | 4.59e-03 |
| 70 | 1.17e-04 | 1.05e-04 | 1.29e-04 | 1.54e-04 | 9.67e-05 | 2.00e-03 |
| 90 | 2.12e-04 | 5.51e-05 | 6.74e-05 | 8.51e-05 | 5.20e-05 | 2.27e-03 |
| 110 | 3.00e-05 | 2.97e-05 | 3.65e-05 | 4.31e-05 | 2.71e-05 | 1.83e-03 |
| 150 | 3.06e-05 | 6.10e-06 | 7.59e-06 | 8.14e-06 | 5.27e-06 | 2.18e-03 |
| 170 | 0.00e+00 | 0.00e+00 | 0.00e+00 | 0.00e+00 | 0.00e+00 | 0.00e+00 |

**Table Rebuttal 1:** relative error on the average concentration at the steady state using different mesh sizes (the mesh size refers to the parameter given as input in Fenics). The error is computed using the finest mesh (N=170) as the reference.

In a similar way, we investigated refining the time step chosen for the Euler scheme. The relative error is presented in **Table Rebuttal 2.** Already with a dt=0.4 the error is of the machine precision or lower where the lactate is the only exception with an error of the order 10^{-5} due to the efflux dynamics.

| Time Step | [GLC]($T$) | [ATP]($T$) | [ADP]($T$) | [GLY]($T$) | [PYR]($T$) | [LAC]($T$) |
|---|---|---|---|---|---|---|
| 0.40 | 1.21e-16 | 0.0 | 0.0 | 0.0 | 1.16e-16 | 3.26e-5 |
| 0.25 | 0.0 | 2.06e-16 | 0.0 | 1.16e-16 | 0.0 | 1.01e-5 |
| 0.18 | 0.0 | 0.0 | 0.0 | 0.0 | 0.0 | 0.0 |

**Table Rebuttal 2:** relative error on the average concentration at the steady state using different time steps (s). The error is computed using the finest time step (dt=0.18 s) as the reference, as for the mesh we verified that by refining the time step the average concentration reached a stable value.

Concerning the image-based geometries, we discussed the choice of the Cut Finite Element Method (CutFEM) to work with complex geometries in our previous work [Farina et al. 2020]. In fact, the classic finite element method (FEM) requires building a mesh that follows the boundaries of, in this case, the astrocyte. CutFEM allows for disentangling the geometry of the astrocyte from the finite element mesh. Thus, we just needed a three-dimensional mesh and embedded the image-based astrocyte in this framework by defining it with a level set function.

To evaluate the geometry-based simulation, the finite element background mesh was built creating a 1-1 mapping between pixel and discretisation after the astrocytic image was post-processed. We assess the quality of the mesh by solving a simple diffusion problem inside the astrocytic shape, for which we obtained the analytical (exact) solution which are compared in **Figure Rebuttal 8**. While the full system can exhibit different convergence, this comparison indicates the right range of our discretization.

**PDE**

$$\begin{cases} \frac{\partial a}{\partial t} = \nabla^2 a + f \\ a(0) = 0 \end{cases}$$

**ODE**

$$\begin{cases} \frac{da}{dt} = f \\ a(0) = 0 \end{cases}$$

**Solution**

$$a(t) = ft$$

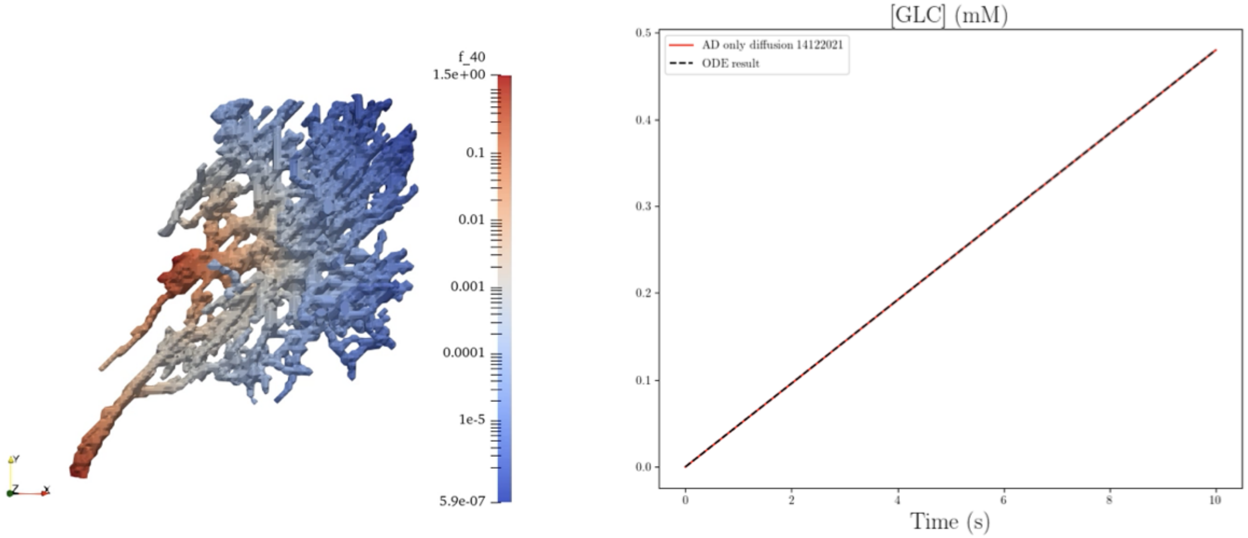


$$|a(T) - a_{exact}(T)| = 2.85e - 09$$

**Figure Rebuttal 7:** In the astrocytic shape we solve the PDE system defined on top, where we know the exact analytical solution. The error evaluated at the final time step between the average concentration inside the astrocyte and the exact solution has an error of the order of 10^{-9}.

Then, considering the healthy control setting presented in the manuscript (Figure 6 A), we investigated the spatial results by refining the time step. We considered two-time steps: dt = 0.08 and dt=0.2, and compared the average concentration of each metabolite at the steady state obtaining an error of the order of 10^{-10}, ensuring that the choice of dt=0.2 was guaranteeing the convergence to the steady state. Additionally, we investigated that also locally the concentration solution reached the same steady state. In **Figure Rebuttal 8,** we show the ATP spatial results in 3D and we compare along a line passing through the astrocyte the ATP for the two time steps and could confirm that they converge.

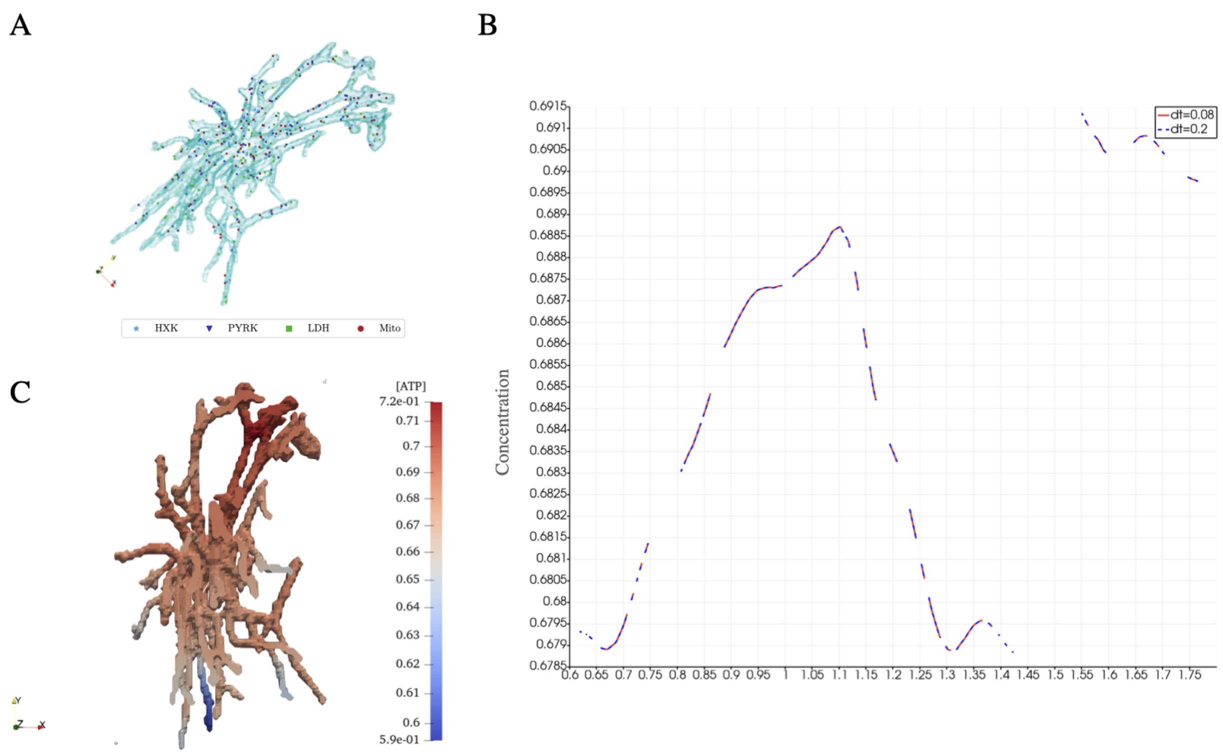


**Figure Rebuttal 8: A)** Setting for the reaction rate sites of control astrocyte **C)** ATP results in 3D **B)** we plot the ATP local concentration with two-time steps (dt= 0.2 and dt=0.08) on the diagonal of the box that contains the astrocyte.

* The authors revise the Discussion. The authors summarize their own results at length, but only marginally compare/discuss their findings with previous observations from the (modelling, experimental and/or clinical) literature. This discussion should be extended and be made more specific.

The discussion has been improved following this remark but also following remarks made by Referee 1 (l.486-493) and Referee 2 on uncertainty quantification ( l.504-507). We added more discussion about other metabolic modelling approaches (l.468-470) and using real geometries in cellular models (l.473-475)

Minor comments:

The authors should consider the following minor points

Thank you for pointing to these minor points!

- L9: While [it] is known … Solved
- L11: Replace "insults" by e.g. trauma Solved

- L36: addressed in [the] literature Solved
- P16 and Fig 5: Inconsistent labelling of subfigures (a-c vs A-C). Solved

We would like to thank the reviewers again for their solid evaluation of our manscript and very valuable suggestions from which the ms has significantly improved. We hope that the reviewers are satisfied and agree with the made ms adaptations.

Sincerely,

Sofia Farina and Alexander Skupin on behalf of all authors
